# Clinical applications of microRNAs

**DOI:** 10.12688/f1000research.2-136.v3

**Published:** 2013-10-08

**Authors:** Per Hydbring, Gayane Badalian-Very

**Affiliations:** 1 Department of Cancer Biology, Dana-Farber Cancer Institute and Harvard Medical School, Boston MA, 02215, USA; 2 Department of Genetics and Medicine, Harvard Medical School, Boston MA, 02115, USA; 3 Department of Medical Oncology, Dana-Farber Cancer Institute and Harvard Medical School, Boston MA, 02215, USA; 4 Department of Medicine, Brigham and Women's Hospital, Boston MA, 02115, USA

## Abstract

MicroRNAs represent a class of small RNAs derived from polymerase II controlled transcriptional regions. The primary transcript forms one or several bulging double stranded hairpins which are processed by Drosha and Dicer into hetero-duplexes. The targeting microRNA strand of the duplex is incorporated into the RNA Induced Silencing Complex from where it silences up to hundreds of mRNA transcript by inducing mRNA degradation or blocking protein translation. Apart from involvement in a variety of biological processes, microRNAs were early recognized for their potential in disease diagnostics and therapeutics. Due to their stability, microRNAs could be used as biomarkers. Currently, there are microRNA panels helping physicians determining the origins of cancer in disseminated tumors. The development of microRNA therapeutics has proved more challenging mainly due to delivery issues. However, one drug is already in clinical trials and several more await entering clinical phases. This review summarizes what has been recognized pre-clinically and clinically on diagnostic microRNAs. In addition, it highlights individual microRNA drugs in running platforms driven by four leading microRNA-therapeutic companies.

## MicroRNA discovery

Two decades have passed since the groundbreaking work from the laboratories of Gary Ruvkun and Victor Ambros, which demonstrated that a small temporal non-coding RNA could influence development of *Caenorhabditis elegans* by base pairing to the 3´ untranslated region (3´ UTR) of a coding messenger RNA (mRNA) thereby regulating its translation ^1, 2^. For several years these RNA molecules were considered to be specific to *C. elegans*. A final recognition that they also played this role in additional systems, including human cells, marked the official birth of microRNAs (miRNAs) ^3– 5^.

miRNAs are separated from other small RNAs by the prediction of a hairpin fold-back structure from the precursor transcript together with expression evidence of an approximately 22 nucleotide-long mature sequence. Currently, 1600 human miRNA precursors have been deposited into miRBase v19 based on analyses of RNA deep-sequencing data ^6^. The nomenclature of these miRNAs is based on a “mir” or “miR” prefix with identifying numbers assigned sequentially at the time of discovery. “mir” denotes a precursor miRNA whereas “miR” denotes a mature miRNA sequence. A precursor miRNA can give rise to one or two mature miRNAs. Similar or identical sequences can be given the same number. Distinction is then accomplished by a letter (similar sequences) or numeral ending (identical sequences). For example, miR-15a and miR-15b have identical 5´ ends but differ by four nucleotides in their 3´ regions. In contrast, miR-16-1 and miR-16-2 are identical but encoded on two separate chromosomes, chromosome 13 and 3, respectively ^7^ miRBase v19.

### Genomic location and transcription of microRNAs

miRNA genes reside either in intergenic regions, within introns of coding or non-coding genes or within exons of non-coding genes ^8^. Approximately one third of miRNAs are intergenic and about one third of all miRNA genomic loci contain clustered miRNAs miRBase v19. Work from the laboratory of David Bartel and others demonstrated that miRNAs that are oriented in the same genomic orientation and separated by less than 50 kb but more than 0.1 kb are highly correlated in their expression patterns. These results suggest that they originated from a miRNA cluster producing polycistronic transcripts ^9, 10^. Consistent with this, the laboratory of David Fisher showed that intronic miRNAs with independent transcriptional start sites were located substantially further away from their host gene start site, displaying a median distance of 57 kb ^11^. Predicting the number of human miRNA clusters and miRNA precursor transcripts using such cutoffs in miRBase generates 175 and 538, respectively.

The majority of miRNAs are transcribed as long primary transcripts by RNA polymerase II and many are capped and polyadenylated ^12, 13^. Analysis of miRNAs residing in intergenic primary transcripts indicates that such transcripts are shorter than protein-coding transcripts with transcriptional start sites about 2 kb upstream of the pre-miRNA and polyadenylation signals 2 kb downstream ^14^. A subset of miRNAs is transcribed by RNA polymerase III. This cluster of miRNAs is located among Alu rich regions on chromosome 19 ^15^.

While most intronic miRNAs seem to be expressed from their host-gene transcriptional machinery, promoters and transcriptional activators of intergenic miRNAs are poorly defined. In order to identify miRNA promoters and their transcription factors in stem cells, the laboratory of Richard Young set up a score system. This system overlaid genomic coordinates of tri-methylated Lysine 4 of Histone 3 (H3K4me3), their proximity to annotated miRNA sequences, expression sequence tag (EST) data and conservation between species with genome-wide association of transcription factors Oct4, Sox2, Nanog and Tcf3. The results demonstrated that miRNAs are regulated by Oct4, Sox2, Nanog and Tcf3 to the same degree as protein-coding genes ^16^. These findings suggest that most miRNAs are transcriptionally regulated in a “protein-coding like” fashion.

### Processing of microRNAs

Primary miRNA transcripts (pri-miRNAs) form hairpin bulges due to sequence complementarity. These hairpins are subject to processing by the Microprocessor complex which takes place in the nucleus ^20^. The Microprocessor consists of the RNase III enzyme Drosha and its partner DiGeorge syndrome critical region gene 8 (DGCR8). Upon binding of DGCR8 to pri-miRNA at the junction of single-stranded to double-stranded RNA, Drosha cuts the pri-miRNA hairpin 11 bp into its double-stranded stem sequence generating slightly smaller hairpins known as pre-miRNAs. Intronic miRNAs are cleaved by Drosha co-transcriptionally preceding splicing of the primary transcript ^17^. Pre-miRNAs are exported into the cytoplasm by the exportin 5 Ran-GTP complex where they are released by GTP hydrolysis and further processed by the RNase III Dicer into an approximate 22 nucleotide duplex. Dicer counts 22 nucleotides from the 5´ end of the pre-miRNA before cleaving, generating a duplex with 3´ end overhangs at each side. RNA helicases facilitate unwinding of the duplex with one strand being incorporated into the targeting miRNA containing RNA Induced Silencing Complex (RISC) together with the Argonaute protein. Thermostability of the 5´ ends determines that the strand will be incorporated in the complex and that the strand is left out will be degraded ^8, 18^.

### MicroRNA functional targeting

The miRNA-RISC complex binds to 3´ UTRs of mRNAs via nucleotide complementarity. Upon binding, it either induces mRNA degradation or inhibits protein translation. It is unclear to what extent mRNA degradation and translational inhibition coincide, but steady state levels suggest that mRNA degradation serves as the major final outcome for miRNA targeted transcripts ^19^. Although binding can occur in 5´ UTRs and open reading frames, targeting in these regions are less frequent and efficient compared to 3´ UTR targeting ^20^. In addition to mRNAs, miRNAs have recently been demonstrated to target other RNA species, including long non coding RNAs (lncRNAs), antisense RNAs and competing endogenous RNAs ^21– 23^. These new layers of functional regulation may certainly add to the clinical value of miRNAs but a deeper description is out of scope for this review.

miRNAs bind their targets through seed- or seed-less pairing. The miRNA “seed” is located in its 5´ end and expands from nucleotides 2–7. miRNAs that are interacting with their targets only through the seed region rarely produce efficient targeting. Additional base pairing of either nucleotide one, eight or both results in canonical seed targeting referred to as “7mers” or “8mers” correlating with functional outcomes. Disrupted complementarity at any of the nucleotides two to seven leads to seed-less targeting. Seed-less targeting depend on compensatory complementarity along the mature miRNA sequence. In contrast, canonical seed targeting may be functional without such additional binding ^20^. Interestingly, a recent paper mapping the human miRNA-mRNA interactome suggests that 60% of seed interactions are non-canonical ^24^. During recent years, a number of prediction algorithms have been developed to track miRNA targeting based on seed pairing, overall complementarity, pairing stability, target site evolutionary conservation and UTR context ^20^.

Generally, a single miRNA is predicted to have hundreds of targets. Prediction algorithms provide significant information for selecting appropriate miRNAs for targets of interest but there are several shortcomings when one seeks the biological outcome of a specific miRNA. First, expression levels of many miRNAs vary greatly between different cell types/tissues or disease states ^10, 25^, and this affects the stoichiometry of the binding of the miRNA to the target, potentially altering its impact. Secondly, the targeting capacity of a certain miRNA can be blunted by the state of its target, for example, shortened 3´ UTRs in cancer cells or point-mutations in the 3´ UTRs ^26, 27^.

The effect of a given miRNA may not necessarily correlate with repression of its targets. 3´ UTRs vary greatly in size, with many harboring binding sites for numerous endogenous miRNAs ^20, 26^. Inhibiting a single miRNA is therefore likely to give non-detectable or very modest derepression of an investigated target. Finally, functional assays demonstrating mRNA or protein alterations preceded by the introduction of ‘mimic miRNAs’ or inhibiting endogenous miRNAs could stem from the execution of indirect biological programs and not necessarily reflect miRNA-to-target interaction.

To combat such issues, miRNA researchers have turned to biochemical methods. Pull-down assays for either the miRNA itself or the RISC-complex followed by RT-PCR, microarray or sequencing could determine if the physical interaction between a given miRNA/mRNA is present or not ^28– 30^. By combining biochemical approaches with functional assays, it is possible to build confidence in specific miRNA targets. Given the huge number of predicted targets for any given miRNA, it is plausible to assume that only a fraction of predicted targets are substantially affected ^31, 32^. Interestingly, a recent study overlaid interacting transcripts with the targets that were significantly downregulated due to exogenous expression of a mimic miRNA in two different cell lines. They observed that the majority of miRNA to transcript interactions may result in no or very weak functional outcomes ^29^. Such combinatory approaches are likely to be crucial in order to fully dissect miRNA functional targeting and they may ease biological network analyses by substantially reducing the number of targets.

## MicroRNAs in disease diagnostics

Observations that miRNAs displayed high stability in paraffin-embedded tissues from clinical samples or in human plasma ^33, 34^ raised the possibility that miRNA expression analysis may be a useful tool to define disease states. An early key report from the laboratory of Todd Golub covering 217 mammalian miRNAs and several hundred samples, including clinical samples, common cancer cell lines and mouse tumors, demonstrated that miRNA profiles could distinguish a tumor’s developmental origin and that miRNAs are generally downregulated in cancers (129 out of 217 miRNAs). Interestingly, poorly differentiated tumors displayed lower miRNA expression compared to tumors with a higher differentiation ^25^. This report was complemented by a study looking at miRNA expression profiles in human solid tumors ^35^. miRNA signatures were further used to define subtypes of cancers, such as the distinction between basal and luminal breast cancers ^36, 37^. Sempere *et al.* showed that ER ^+^PR ^+^HER2 ^+^, ER ^-^PR ^-^HER2 ^+^ and ER ^-^PR ^-^HER2 ^-^ breast cancer tumors exhibited distinct miRNA patterns with expression of miR-205 in triple negative breast cancers correlating positively with clinical outcome. Analysis of separate cancers demonstrated miRNA profiles could predict clinical progression ^38, 39^. miR-15a and miR-16-1 act as prognostic biomarkers in chronic lymphocytic leukemia (CLL) and let-7a is a marker for lung cancers ^38, 39^. The laboratory of Tyler Jacks gave an ultimate support to the notion that the majority of miRNAs display lower expression in tumors and therefore may play tumor suppressive properties. By using conditional knockout mice for Dicer crossed with a K-Ras driven lung cancer model they demonstrated that a global reduction of miRNA biogenesis leads to reduced survival in affected mice ^40^.

In addition to cancer, miRNA expression profiles could also be used to distinguish distinct forms of heart disease ^41^, muscular disorders ^42^ and neurodegenerative diseases ^43^. The human miRNA-associated disease database ( HMDD) serves as a resource for scientists screening the constantly increasing number of miRNA profiles for a wide range of diseases ^44^. At the time of writing this review, the HMDD-database covered disease-associations from 2741 scientific publications.

### Circulating microRNAs

The vast majority of miRNA expression profile reports stem from solid tissues although miRNAs can be readily detected in human plasma, serum or total blood due to their small size and high stability ^35, 45^. The potential of circulating miRNAs as biomarkers in serum was indicated early by studies examining patients with diffuse large B-cell lymphoma, highlighting miR-21 as a potential biomarker ^46^. Prostate cancer patients could be distinguished from healthy counterparts by analyzing the expression level of miR-143 ^35^. These studies were followed by additional reports of miRNAs in breast cancer (using whole blood) ^47^, colorectal cancer (using plasma) ^48^ and squamous cell lung cancer (using sputum) ^49^ patients. Interestingly, Boeri *et al.* demonstrated that circulating miRNAs may also be used for predicting purposes. They displayed miRNA signatures with strong predictive value in lung cancer patients years before the onset of disease by analyzing expression in samples taken before diagnosis, at the time of detection by computed tomography and in disease-free smokers ^50^.

In a massive undertaking Keller *et al.* analyzed 863 miRNAs from 454 human blood samples. The samples were from patients suffering from 14 separate diseases including lung cancer, prostate cancer, pancreatic ductal adenocarcinoma, melanoma, ovarian cancer, gastric tumors, Wilms tumor, pancreatic tumors, multiple sclerosis, chronic obstructive pulmonary disease, sarcoidosis, periodontitis, pancreatitis and myocardial infarction. On average, more than 100 miRNAs were deregulated in the blood for each disease. By utilizing this data and developing mathematical algorithms and probability plots the authors were able to accurately predict the disease in more than two thirds of individuals involved in the study ^51^. It should be noted that miRNA patterns in blood are unlikely to be the same between different types of blood cells. Distinct hematopoietic lineages display different miRNA-profiles ^52^, possibly suggesting that differences in expression in specific diseases are due to shifts in hematopoietic cell populations. This could be pronounced in diseases directly affecting the blood such as cancers spreading to the bone marrow or multiple sclerosis, a chronic central nervous system disease associated with an abnormal immune system response. Using 27 distinct cell populations with the highest variance, Keller *et al.* computed that such shifts could account for a maximum of 60% of differences in the observed miRNA profiles ^51^.

How are miRNAs released into the blood stream? Rechavi *et al.* demonstrated that synthetic miRNA mimics, viral miRNAs or endogenous miRNAs could be released from B-cells and taken up by T-cells upon cell contact ^53^. Further, Pegtel *et al.* demonstrated secretion of miRNAs from Epstein-Barr virus (EBV)-infected B-cells via exosomes, providing protection of the miRNA from RNases ^54^ and Yuan *et al.* reported the transfer of miRNAs from embryonic stem cells to fibroblasts via embryonic stem cell microvesicles ^55^. Finally, Kosaka *et al.* demonstrated that miRNA secretion occurs in a ceramide-dependent fashion, which could be blunted by knocking down nSmase2, an enzyme required for ceramide biosynthesis ^56^.

In addition to serum/plasma, the presence of miRNAs has also been demonstrated in urine ^57^ and saliva ^58^, with two miRNAs, miR-125a and miR-200a, displaying lower expression in patients with oral squamous cell carcinoma compared to healthy subjects ^59^.

### MicroRNA profiling methods

miRNA expression profiling and disease association studies are conducted using a set of different methods including miRNA microarray platforms, quantitative real-time polymerase chain reaction (qRT-PCR), *in situ* hybridization and high throughput sequencing. Both qRT-PCR and hybridization methods are highly sensitive and quantitative. This makes them useful for analyzing small sets of miRNAs ^60^. It is worth noting that there are limitations with hybridization methods due to potential hybridization of the probe to pri-, pre- and mature miRNAs. Detection of mature miRNA sequences through qRT-PCR demands a stem-loop RT primer, specific primers for amplification of the cDNA and a TaqMan probe (Roche Molecular Diagnostics, Applied Biosystems) ^61^. *In situ* hybridization can either be represented by fluorescence *in situ* hybridization (FISH) or chromogenic *in situ* hybridization (CISH) and detection of miRNAs as well as other non-coding RNAs is possible without protease-treatment of tissues ^62^.

For high-throughput studies, miRNAs are profiled using array platforms or sequencing. Next-generation sequencing technologies offer lower costs and shorter processing time making this platform highly attractive. Today, researchers frequently use sequencing techniques to profile miRNA signatures in distinct sets of tissues/diseases.

### Clinical microRNA diagnostics

A major challenge in clinical diagnostics is cancers with poor differentiation. Even though these cancers account for only a few percent of malignancies, they display substantially distinct gene-signatures making it notoriously hard to trace the cell of origin despite the availability of the latest microRNA platforms ^63^.

Perhaps the most exciting potential of miRNAs in current diagnostics started with a study comparing miRNA expression using miRNA microarrays in 205 primary versus 131 metastatic tumors covering 22 different tumor origins. The authors developed a binary decision tree classifier based on miRNA expression with tissues displaying the highest specificity of certain miRNAs placed at the top of the tree. Following this classifier, a remarkably low number of 48 miRNAs predicted tissue origin at close to 90% accuracy when tested on a blinded set ^64^ ( Table 1). This study, together with subsequent studies confirming the accuracy of using miRNAs as diagnostics for tumors of unknown origin ^65, 66^, was partly driven by the miRNA diagnostics company Rosetta Genomics, Israel.

**Table 1.** Rosetta Genomics initial binary tree classifier consisting of 24 nodes giving rise to 25 leaves or defined cancer types. Each node uses 1 to 5 miRNAs for classification. Nodes giving no defined cancer types make broader distinctions. For example, Node #3 distinguishes epithelial tissues from mixed origin. Table is adapted from Rosenfeld *et al.* 2008.

*Node*	*MicroRNAs used at node*	*Defined cancer types at node*
1	miR-122a, miR-200c	Liver
2	miR-372	Testis
3	miR-200c, miR-181a, miR-205	–
4	miR-146a, miR-200a, miR-92a	–
5	miR-142-3p, miR-509	Lymph node, Melanocytes
6	miR-92b, miR-9*, miR-124a	Brain
7	miR-152, miR-130a	Meninges
8	miR-205	Thymus (type B2)
9	miR-192, miR-21, miR-210, miR-34b	–
10	miR-194, miR-382, miR-210	Lung-pleura, Kidney
11	miR-187, miR-29b	Sarcoma, Gastro Intestinal Stromal Tumors
12	miR-145, miR-194, miR-205	–
13	miR-21, let-7e	Lung (carcinoid)
14	let-7i, miR-29a	Colon
15	miR-214, miR-19b, let-7i	Stomach, Pancreas
16	miR-196a, miR-363, miR-31, miR-193a, miR-210	–
17	miR-27b, let-7i, miR-181b	Breast, Prostate
18	miR-205, miR-141, miR-193b, miR-373	–
19	miR-106b, let-7i, miR-138	Thyroid
20	miR-10b, miR-375, miR-99a	–
21	miR-205, miR-152	Lung, Bladder
22	miR-345, miR-29c, miR-182	Endometrium, Ovary
23	miR-192, miR-345	Thymus (type B3)
24	miR-182, miR-34a, miR-148b	Lung (squamous), Head and Neck

Based on these reports, Rosetta Genomics is now offering a panel (miRview-mets2) to clinicians so that the origin of metastatic cancers can be identified where the primary origin of metastasis is questionable. The panel consists of a test of 64 miRNA biomarkers validated on 489 samples of which 146 represent metastatic tumors covering 42 tissues of origin ^67^. In addition to the “miRview-mets2” panel, Rosetta Genomics also offers four additional clinical tests: “miRview-lung”, “miRview-squamous”, “miRview-meso” and “miRview-kidney”. MiRview-lung differentiates four types of primary lung cancer (small cell lung cancer, squamous non-small cell lung cancer (NSCLC), non-squamous NSCLC and carcinoid) using eight separate miRNA biomarkers ^68^. MiRview-squamous separates non-small cell lung cancers (NSCLC) into squamous cell carcinomas and adenocarcinomas using a single miRNA, miR-205 ^69^. MiRview-meso defines samples into a mesothelioma or non-mesothelioma origin based on three separate miRNAs ^70^ and miRview-kidney separates kidney cancer into its four primary types (benign oncocytoma, clear cell renal carcinoma, papillary renal carcinoma and chromophobe renal carcinoma) using six miRNAs. All clinical panels rely on the same tree-classifier as in the original paper ^63, 71^.

The move of miRNA diagnostics into the clinics led by Rosetta Genomics and others may aid the use of personalized medicine in the treatment of cancers. Importantly, miRNA diagnostics are not just an additional approach for differentiated cancers but a potential breakthrough for cancers initially defined as of unknown origin.

### Nucleotide polymorphism in diagnostics

In addition to more well-established approaches of analyzing miRNA expression profiles, predisposition to certain cancer types may be predicted by the existence of single nucleotide polymorphisms (SNPs). SNPs can reside in either precursor miRNAs, mature miRNAs or in 3´ UTRs disrupting or creating miRNA binding sites ^72^.

Screening of 42 miRNA expression profiles in chronic lymphocytic leukemia (CLL) revealed the presence of mutations in 5 miRNAs; all SNPs were detected in the pri- or pre-miRNA structure. For one of these, miR-16-1, a mutation in the precursor transcript led to a significant reduction in mature miRNA expression ^40^. Other reports of SNP variants in miRNA precursors where the mutant variant correlates with an increased risk of cancer include miR-196a-2 and miR-499 in breast cancer ^73^, miR-196a-2 in head and neck cancer ^74^, whilst increased mature levels of miR-146a can lead to an earlier onset of breast and ovarian cancers ^75^. In addition, SNPs affecting factors in the miRNA processing machinery lead to either an increased or reduced risk of renal cell carcinoma depending on factors which were affected ^76^.

Examples of SNPs located in 3´ UTRs of miRNA target sites include the binding site of miR-221/222 and miR-146 in Kit ^77^ as well as the let-7 binding site in the 3´ UTR of K-Ras. The latter displays an alteration associated with an increased risk of NSCLC ^28^. Further, a SNP in the miR-184 binding site in the 3´ UTR of tumor necrosis factor alpha-induced protein 2 (TNFAIP2) resulted in an increased risk of squamous cell carcinoma of the head and neck ^78^.

Using available SNP data from “The 1000 Genomes Project” ^79^, Richardson *et al.* concluded that approximately 5% of all SNPs maps to miRNA recognition elements (MREs). Among these SNPs, 22% associated with disease phenotype ^80^. Interestingly, these numbers suggest that SNPs residing within MREs are likely to be under selective pressure.

## MicroRNAs in therapeutics

The idea of using miRNAs in therapeutics is highly appealing after observing the outcomes from manipulating these molecules. Rather than intercepting a single target as in the case of selective protein inhibitors, miRNAs can modulate entire gene programs. Importantly, the outcome is tuning of target expression instead of blunting it which should be less detrimental to healthy tissues. Given the notion of deregulation of these molecules in a wide range of diseases and the robust degree of accurate profiling, one would estimate the chances of mapping specific miRNAs for manipulation of a given disease with minimal side-effects to be fairly good.

**Figure 1.**
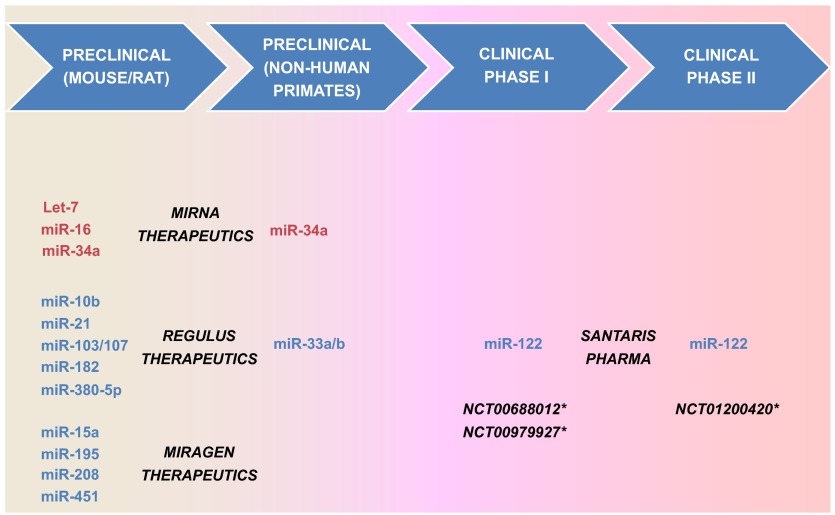
Leading companies developing microRNA therapeutics. The four stages at the top denote the progress of individual miRNA drugs. Red colored miRNAs indicate replacement therapy with drug mimicking the endogenous miRNA. Blue colored miRNAs indicate inhibition therapy with drug targeting the endogenous miRNA. *Santaris Pharma has completed two Phase I clinical trials and is currently in Phase II with their miR-122 targeting drug miravirsen.

miRNAs fall into the class of RNAi-based therapeutics, a concept that is not completely new, having given birth to more than 20 clinical trials so far ^81^, but unlike short interfering RNAs/short hairpin RNAs (siRNAs/shRNAs), which are designed to target a single transcript, modulation of miRNAs, will affect hundreds of transcripts and so would potentially be capable of shutting down entire deregulated pathways. Of course, specific targeting of disease associated transcripts is probably required since a completely random modulation of hundreds of transcripts would be too harmful for the patient. However, such issues can be relatively easily investigated from *in vitro* studies conducting genome-wide mRNA expression together with pathway enrichment analysis. To date there is only one miRNA drug in clinical trials (SPC3649: inhibitor/antagomir of miR-122, Santaris Pharma, Denmark) ( Figure 1). The slow progress stems from the general technical challenges with RNAi-based therapeutics including delivery, stability and avoidance of activating immune responses.

As discussed earlier in this review, endogenous miRNAs remain highly stable even when secreted into circulation. They resist nucleases by being enclosed into microvesicles or exosomes. Creating complex delivery-vehicles for miRNA therapeutics is feasible but could be time-consuming and costly. To minimize stability issues, a range of chemical modifications have been developed for siRNAs, modifications likely to be transferrable to miRNAs due to their similar structures. These include Phosphorothioate, 2´-O-methyl RNA, 2´-Fluoro-RNA and 2´O-methoxy-ethyl RNA, all of which provide greater nuclease resistance ^81, 82^. A phosphorothioate modification exchanges a nonbridging oxygen atom with a sulfur atom at the phospho-backbone of the unmodified RNA. In addition to providing nuclease resistance this also promotes RNase H-mediated cleavage of targeted transcripts. The 2´O-methyl modification adds a methyl group (-CH _3_) to the second carbon of the ribose. Likewise, 2´Fluoro adds a fluorine atom at this position and 2´O-methoxy-ethyl adds a methoxy-ethyl group (-C _3_H _7_O). All 2´-modifications increase Watson-Crick base pairing to targeted transcripts and substantiate nuclease resistance due to closer proximity between the 2´ group and 3´ phospho-group. In addition, a modification known as locked nucleic acid (LNA) is widely used in synthesis for miRNA inhibiting drugs, sometimes referred as antagomirs. This modification utilizes a bridge between the 2´O group and 4´ carbon atom also referred to as 2´O-4´C-methylene linked ribonucleotides ^82^.

Even though siRNAs, and in a similar way miRNA mimics, can be stabilized through chemical modifications, their size still demands complex vehicles in order to ensure delivery to different tissues *in vivo* ^83^. In contrast, miRNA inhibiting drugs, or antagomirs, can be synthesized as short single-stranded oligonucleotides, their small size together with their potency/stability provided by the LNA-modifications make delivery possible without vehicle-systems. For the rest of this review we will summarize miRNA drugs currently in clinical trials or likely to enter clinical trials in the near future. We bring up the platforms of four leading miRNA therapeutic companies of which three utilize the antagomir technique.

### Miravirsen (Santaris Pharma)

Miravirsen (or SPC3649) is a LNA-modified oligonucleotide designed to inhibit miR-122 developed by Danish firm Santaris Pharma A/S ^84^. This liver specific and highly abundant miRNA accounts for more than 70% of all miRNAs in the liver and has been shown to be crucial for the functional infection of Hepatitis C virus (HCV). The exact mechanism of how this miRNA facilitates viral replication is not clearly understood. It is suggested that interaction between miR-122 and two seed-sites in the 5´ noncoding region of HCV induces viral transcripts, giving this miRNA a non-classical function of inducing rather than inhibiting the function of its target ^85^.

Treatment of chronic Hepatitis C-infected chimpanzees with miravirsen led to suppression of HCV without any obvious side-effects. Further, liver transcriptome analysis revealed 259 mRNAs containing full 8mer-seed miR-122 predicted binding sites to display increased expression following treatment, thus indicating such transcripts to be targeted by endogenous miR-122 in Hepatitis C. It is likely that some of those transcripts play additional therapeutic roles. Total serum cholesterol was reduced with downregulation of cholesterol metabolism genes ^84^.

Since reporting their non-human primate study, miravirsen has gone through two phase I clinical trials, successfully proving that the drug is safe even in humans ( NCT00688012, NCT00979927), and a Phase IIa clinical trial ( NCT01200420) ( Figure 1). This Phase IIa trial enrolled 38 patients with treatment-naïve chronic HCV infection to monitor safety, tolerability, pharmacokinetics and efficacy on HCV viral titer. Multiple dosage of miravirsen administered subcutaneously to patients gave promising outcomes with a mean reduction of HCV RNA levels by two to three logarithmic levels. Further, almost half of the patients treated by the highest dose displayed undetectable levels of HCV RNA within 4 weeks. These results are encouraging and highlight miravirsen as a potential future replacement therapy for patients with chronic HCV infection.

Discovering the general potential of miRNA inhibitory therapeutics using LNA-based or similar platforms for a wide range of diseases is a major task for the future. One might imagine that the tissue specificity of miR-122, its high abundance in this tissue compared to other miRNAs and the general convenience of delivering drugs systemically to the liver makes inhibition in this case easier than most other miRNAs. Despite such possible concerns, the development of miravirsen by Santaris Pharma provides a landmark breakthrough for miRNA based therapeutics.

### Anti-miRs (Regulus Therapeutics)

Regulus Therapeutics is a San Diego based company with a wide-range program focusing on targeting endogenous miRNAs through inhibitory oligonucleotides in hepatocellular carcinoma, kidney fibrosis, atherosclerosis, HCV infection and glioblastoma. To keep such a platform running, Regulus has partnered with the corporate giants Sanofi, GlaxoSmithKline and AstraZeneca. In 2010, Regulus signed a deal with GlaxoSmithKline for the development of a miR-122 inhibitor drug, thus providing direct competition with Santaris Pharma (see above).

None of Regulus’ programs have reached clinical phases but substantial preclinical work has been completed including a non-human primate study of inhibiting miR-33a/b for the treatment of atherosclerosis ^86^ ( Figure 1). miR-33b and miR-33a are encoded in the introns of the transcription factor loci *SREBP1* and *SREBP2*, respectively, and are involved in the regulation of cholesterol and fatty acid homeostasis. This puts them as potential targets for treatment of cardiovascular diseases ^86^. By treating African Green monkeys subcutaneously with 2´-fluoro-methoxyethyl-phosphorothioate modified antisense-miR-33 oligonucleotides (anti-miR-33), the study demonstrated a decrease in very-low-density lipoprotein (VLDL)-triglycerides and an increase in high-density lipoprotein (HDL) ^86^. Mechanistically, there was a significantly reduced repression of miR-33 predicted target genes and primates went through treatment without displaying significant side-effects ^86^.

Using another anti-miR (anti-miR-21), Regulus convincingly demonstrated that their technology could be used for the inhibition of migration/invasion of glioma in mice ^87^, attenuating cardiac dysfunction in a mouse model of cardiac disease ^88^ and executing an anti-fibrotic response in mice exposed to kidney injury ^89^. Results from additional mouse-models exposed to Regulus-developed anti-miRs include suppression of lung-metastasis originating from breast tumors (anti-miR-10b) ^90^, inhibition of neuroblastoma (anti-miR-380-5p) ^91^, antagonizing liver metastasis sourcing from melanoma (anti-miR-182) ^92^ and improved glucose homeostasis and insulin sensitivity (anti-miR-103/107) ^93^ ( Figure 1).

### MicroRNA replacement therapy, MRX34 (Mirna Therapeutics)

In contrast to the companies described above, Mirna Therapeutics is not focusing their platform around the inhibition of endogenous miRNAs but instead on introducing synthetic miRNA mimics as replacement therapies. The idea is to restore expression of certain miRNAs in tumors to a comparable level to surrounding healthy tissues. The platform of Mirna Therapeutics consists of eight drug candidates, solely focused on addressing tumor suppressive roles of miRNAs. Three out of eight candidate mimics represent miRNAs demonstrated by numerous publications to display tumor suppressive properties (miR-34, let-7 and miR-16). Their lead candidate, MRX34, a miR-34a mimic compound, will probably be the first miRNA replacement compound to reach clinical trials ( Figure 1). At the time of writing this review, Mirna Therapeutics is recruiting participants to a Phase I study of MRX34 ( NCT01829971).

miR-34a represents one of the most documented tumor suppression-associated miRNAs, being a transcriptional product of the transcription factor and genome guardian p53 ^94^. Pre-clinical work by Mirna Therapeutics has demonstrated potent anti-tumor effects by introducing miR-34a mimics into a variety of mice cancer models ^95, 96^.

The usage of miRNA mimics for systemic delivery is challenging compared to anti-miR drugs. miRNA mimics need to be double-stranded in order to be processed correctly by the cellular RNAi-machinery and therefore cannot be administered “naked”. Successful delivery therefore requires complex delivery vehicles mimicking physiological settings where miRNAs reside in microvesicles or exosomes. For MRX34, Mirna therapeutics has developed custom nanoparticle liposomes. According to company information ( MIRNA THERAPEUTICS) these liposomes increase stability, enhance delivery and prevent immune response effects. Extensive pre-clinical testing of MRX34 in mouse models of hepatocellular carcinoma using liposomes provided promising outcomes and the upcoming clinical trial is recruiting patients with non resectable primary liver cancer or metastatic cancer with liver involvement ( NCT01829971).

### Anti-miRs (miRagen Therapeutics)

miRagen Therapeutics is an American company founded in 2007 for the development of miRNA based drugs treating cardiac and muscular diseases. A partner alliance with Danish Santaris Pharma gives miRagen commercial rights to the LNA-based technique developed by Santaris. miRagen has programs covering both anti-miR and miRNA mimics. The anti-miR program contains candidate drugs relatively close to clinical stages ( Figure 1). This platform includes three drugs: MGN-9103, MGN-1374 and MGN-4893.

The lead drug, MGN-9103, targets miR-208 with implications for the treatment of chronic heart failure. miR-208 is a heart specific miRNA located in an intron of the alphaMHC gene, which has been demonstrated to be required for cardiac hypertrophy, myosin switching and fibrosis in response to stress ^97^. Such cardiac remodeling was blocked by treating rats subcutaneously with LNA-based anti-miR-208 during hypertension induced heart failure ^98^. Interestingly, MGN-9103 was also recently suggested to play beneficial roles in a mouse model of diabetes/obesity ^99^.

MGN-1374 targets miR-15 and miR-195, miRNAs shown to be upregulated in mouse hearts shortly after birth. Upregulation of miR-15 and miR-195 executes a postnatal cell cycle arrest during the process of heart regeneration after myocardial infarction ^100^. By inhibiting these miRNAs using their LNA-based MGN-1374, miRagen enables post-myocardial infarction remodeling. Such remodeling enhanced heart function and induced cardiomyocyte proliferation in mice and pigs ^101^.

MGN-4893, targets miR-451, a miRNA required for the expansion of red blood cells. Inhibition of miR-451 in mice using anti-miR-451 blocked erythrocyte differentiation suggesting that such inhibitors could be useful for the treatment of disorders leading to abnormal red blood cell production such as polycythemia vera ^102^.

## Concluding remarks

MicroRNAs have come a long way since the initial discoveries two decades ago. Their emerging potential as biomarkers in clinical diagnostics as well as modulators for the treatment of a variety of diseases is truly exciting. In the near future it will become clearer as to whether they have the power to become established as new molecular diagnostic benchmarks and whether microRNA-based therapy can compete with that of selective protein inhibitors.
