# Supplementary material for: Clinical applications of microRNAs
Source: F1000Res. 2013 Oct 8;2:136. Originally published 2013 Jun 6. [Version 3] doi: 10.12688/f1000research.2-136.v3 (PMC3917658; doi:10.12688/f1000research.2-136.v3)
Supplement: Peer review history

# Peer review: Referee response for version 2

**Published:** 2013-09-16

**Authors:** Gustavo Gutierrez Gonzalez

**Affiliations:** 1 Vrije Universiteit Brussel, Brussels, Belgium

The authors have satisfactorily addressed all my previous comments. This is a very informative and brilliantly written review on miRNAs and their current and potential use in human diseases' diagnosis and therapy. I only have two more minor suggestions to finalize the review:

1. Page 3 of 14: When the authors cite and discuss reference 37 (Sempere *et al*.) the number '2' is missing from one of the HER2 genotypes they mentioned. Also, when looking to the original paper published in 2007, I am not sure the authors refer to the right TMA genotypes, and their conclusions seem a bit too vague: what does it mean that miR-145 shows an "opposite pattern"? That statement is not that clear to me when I look at Figure 3 of the original paper.
2. Page 8 of 14: The authors talk about "heart generation" but it should read "heart regeneration". I would suggest: "Upregulation of miR-15 and miR-195 executes a postnatal cell cycle arrest during the process of heart regeneration after myocardial infarction (ref. 100)".

I have read this submission. I believe that I have an appropriate level of expertise to confirm that it is of an acceptable scientific standard.

# Peer review: Referee response for version 1

**Published:** 2013-06-24

**Authors:** Gustavo Gutierrez Gonzalez

**Affiliations:** 1 Vrije Universiteit Brussel, Brussels, Belgium

Hydbring and Badalian-Very summarize in this review, the current status in the potential development of clinical applications based on miRNAs’ biology. The article gives an interesting historical and scientific perspective on a field that has only recently boomed; focusing mostly on the two main products in the pipeline of several biotech companies (in Europe and USA) which work with miRNAs-based agents, disease diagnostics and therapeutics. Interestingly, not only the specific agents that are being produced are mentioned, but also clever insights in the important cellular pathways regulated by key miRNAs are briefly discussed.

Minor points to consider in subsequent versions:

1. Page 2; paragraph *‘Genomic location and transcription of microRNAs’:* the concept of miRNA clusters and precursors could be a bit better explained.
2. Page 2; paragraph *‘Genomic location and transcription of microRNAs’:* when discussing the paper by the laboratory of Richard Young (reference 16); I think it is important to mention that that particular study refers to stem cells.
3. Page 2; paragraph *‘Processing of microRNAs’:* “Argonate” should be replaced by “Argonaute”.
4. Page 3; paragraph *‘MicroRNAs in disease diagnostics’:* are miR-15a and 16-1 two different miRNAs? I suggest mentioning them as: miR-15a and miR-16-1 and not using a slash sign (/) between them.
5. Page 4; paragraph *‘Circulating microRNAs’:* I am a bit bothered by the description of multiple sclerosis (MS) only as an autoimmune disease. Without being an expert in the field, I believe that there are other hypotheses related to the etiology of MS.
6. Page 5; paragraph *‘Clinical microRNA diagnostics’:* Does ‘hsa’ in hsa-miR-205 mean something?
7. Page 5; paragraph *‘Clinical microRNA diagnostics’:* the authors mention the company Asuragen, Austin, TX, USA but they do not really say anything about their products. I suggest to either remove the reference to that company or to include their current pipeline efforts.
8. Page 6; paragraph *‘MicroRNAs in therapeutics’:* in the first paragraph the authors suggest that miRNAs-based therapeutics should be able to be applied with “minimal side-effects”. Since one miRNA can affect a whole gene program, I found this a bit counterintuitive; I was wondering if any data has been published to support that statement. Also, in the same paragraph, the authors compare miRNAs to protein inhibitors, which are described as more specific and/or selective. I think there are now good indications to think that protein inhibitors are not always that specific and/or selective and that such a property actually could be important for their evidenced therapeutic effects.
9. Page 6; paragraph *‘MicroRNAs in therapeutics’:* I think the concept of “antagomir” is an important one and could be better highlighted in the text.
10. Throughout the text (pages 3, 5, 6, and 7): I am a bit bothered by separating the word “miRNA” or “miRNAs” at the end of a sentence in the following way: “miR-NA” or “miR-NAs”. It is a bit confusing considering the particular nomenclature used for miRNAs. That was probably done during the formatting and editing step of the paper.
11. I was wondering if the authors could develop a bit more the general concept that seems to indicate that in disease (and in particular in cancer) the expression and levels of miRNAs are in general downregulated. Maybe some papers have been published about this phenomenon?

I have read this submission. I believe that I have an appropriate level of expertise to confirm that it is of an acceptable scientific standard.

# Response

**Published:** 2013-08-15

**Authors:** Per Hydbring

**Affiliations:** Dana-Farber Cancer Institute and Harvard Medical School, USA

We thank reviewer Dr. Gustavo Gutierrez Gonzalez, for very valuable comments helping to improve our manuscript. We have adjusted our text according to the comments from Dr. Gutierrez Gonzalez. For clarification, we include a bullet-point response to each comment below.

1. In response to this comment we have added a few words in this paragraph. Further, we added a sentence in the previous paragraph “MicroRNA discovery”.
2. We have clarified in this paragraph that the study refers to stem cells.
3. We thank the reviewer for pointing this out. This typo has been adjusted.
4. miR-15a and 16-1 originate from the same polycistronic transcript and have identical seed sequences. However, as the reviewer point out, they are still two separate mature miRNAs. We have therefore adjusted the text according to the reviewer’s suggestion to avoid confusion.
5. We agree with the reviewer of this misleading description and have adjusted the text accordingly.
6. This means “homo sapiens” but to avoid confusion we have removed it from miR-205.
7. In accordance to the reviewer’s suggestion we have removed this company reference.
8. Since there is currently only one miRNA drug in clinical trials, there is little direct evidence of that miRNAs can be applied with minimal side-effects. In support of our hypothesis, their mechanism of action is tuning expression rather than blunting their targets which reasonably should be less detrimental to healthy tissues. We have added one sentence in this paragraph to clarify our hypothesis. We further agree with the reviewer that a lot of enzymatic protein inhibitors could be more selective. However, the purpose of these drugs is selectivity. In contrast, miRNA drugs are developed with the idea of controlling multiple gene-components in the same or overlapping signaling-pathways. Such gene-products are not limited to proteins with enzymatic activity but could include any deregulated genes or proteins in a given disease.
9. We agree with the reviewer and have made slight adjustments in the text to better highlight this concept.
10. This has been an editing/formatting mistake. In our original manuscript there are no separations of the word “miRNA” or “miRNAs”.
11. We describe downregulation of miRNAs in the section “MicroRNAs in disease diagnostics” referring to a few publications (25, 35-39). To better clarify a general impact of reduced miRNA expression in cancers we included an additional reference (40), describing a conditional knockout mice model for miRNA biogenesis leading to reduced survival in affected mice.

# Peer review: Referee response for version 1

**Published:** 2013-06-18

**Authors:** Florian Karreth

**Affiliations:** 1 Medicine, Beth Israel Deaconess Medical Center, Boston, MA, USA

This review article by Hydbring and Badalian-Very summarizes our current knowledge of microRNAs and outlines their clinical value. The authors provide a comprehensive overview of microRNA biology and target prediction and discuss the utility of miRNAs as prognostic and diagnostic markers and therapeutic targets. Finally, Hydbring and Badalian-Very highlight the various strategies adopted by leading biotechnology companies and the state of their current clinical trials. 

I have two minor suggestions regarding the biology of miRNAs that the authors may wish to include in their manuscript: 

1. A recent paper by paper by Tollervey and colleagues ( Helwak *et al*., Cell 2013) describes that a large fraction of miRNAs bind their targets independent of seed match complementarity at nucleotides 2-7. This observation greatly affects our ability to accurately predict miRNA targets using existing algorithms.
2. Besides the 3’UTR, miRNAs have been demonstrated to target the 5’UTR and coding sequence of mRNAs as well as other RNA species such as lncRNAs, pseudogenes, rRNAs and tRNAs.

I have read this submission. I believe that I have an appropriate level of expertise to confirm that it is of an acceptable scientific standard.

# Response

**Published:** 2013-08-15

**Authors:** Per Hydbring

**Affiliations:** Dana-Farber Cancer Institute and Harvard Medical School, USA

We thank reviewer Dr. Florian Karreth for very valuable comments helping to improve our manuscript. We have adjusted our text according to the comments from Dr. Karreth. For clarification, we include a bullet-point response to each comment below.

1. We thank the reviewer for pointing out this paper which brings the understanding of microRNA targeting to a higher level. We have included this reference and commented on it in the text (paragraph “MicroRNA functional targeting”).
2. The reviewer makes a very important point and to cover some of these mechanisms we have added three references and commented shortly on them in the text (paragraph “MicroRNA functional targeting”). However, since our main focus is on the clinical aspects of microRNAs we believe a deeper description of non-canonical microRNA targeting is out of scope for this review.
